# The Association Between Systemic Inflammatory and Metabolic Indices and Early Adverse Clinical Outcomes in Adult Patients Admitted to the Post-anaesthesia Care Unit: A Retrospective Observational Study

**DOI:** 10.4274/TJAR.2026.262462

**Published:** 2026-06-26

**Authors:** Bedirhan Günel, Ayşe Şencan, Zeynep Yasemin Tavşanoğlu, Fatihhan Zeytun, Ceren Altıntaş, Elif Rana Kılıç, Betül Erdemir, Ayşe Zeynep Turan Cıvraz

**Affiliations:** 1 University of Health Sciences Türkiye Kocaeli City Hospital, Clinic of Anaesthesiology and Reanimation, Kocaeli, Türkiye

**Keywords:** Blood urea nitrogen to albumin ratio, monocyte to lymphocyte ratio, inflammation, post-anaesthesia care unit, postoperative care

## Abstract

**Objective:**

This study aimed to investigate the association of systemic inflammatory and metabolic indices with short-term adverse clinical outcomes in adult patients admitted to the post-anaesthesia care unit (PACU).

**Methods:**

In this retrospective single-center observational study, adults admitted to the PACU following surgical procedures were analysed. The association between systemic inflammatory and metabolic indices, calculated from routine laboratory data, and short-term clinical outcomes was evaluated. Missing data were handled using multiple imputation. Associated risk factors were examined using multiple statistical analyses, and ridge regression was performed to assess model stability and potential multicollinearity. Receiver operating characteristic (ROC) analysis was performed to assess the discriminative ability of the investigated markers.

**Results:**

A total of 860 patients were included in the analysis. In multiple analyses, use of inotropic agents on arrival at the PACU, higher monocyte-to-lymphocyte ratio (MLR), and higher blood urea nitrogen-to-albumin ratio were associated with poor short-term clinical outcomes. ROC analysis demonstrated limited discriminative performance, with area under the curve values of 0.545 for the MLR and 0.582 for the blood urea nitrogen-to-albumin ratio in predicting poor short-term clinical outcomes.

**Conclusion:**

Higher monocyte-to-lymphocyte and blood urea nitrogen-to-albumin ratios were associated with poor short-term clinical outcomes in adult patients admitted to the PACU. However, the low discriminative performance observed in ROC analyses suggests that these indices should not be interpreted as strong standalone predictors. Instead, they may provide supporting information within multiparametric clinical risk assessment models.

**Main Points**

• Systemic inflammatory and metabolic indices derived from routine laboratory data were evaluated in adult patients admitted to the post-anaesthesia care unit after surgery.

• Higher monocyte-to-lymphocyte and blood urea nitrogen-to-albumin ratios were associated with poor short-term clinical outcomes.

• Receiver operating characteristic analyses showed limited discriminative performance for these indices.

• These markers may reflect aspects of postoperative physiological stress and should be interpreted cautiously within the broader clinical context.

## Introduction

The postoperative period is a phase during which physiological changes related to surgery and anaesthesia subside, and patients’ clinical stability is reassessed, making close monitoring essential.^1^ Post-anaesthesia care units (PACUs) are specialised care areas designed to closely monitor fundamental physiological parameters such as respiratory, cardiovascular, and neurological status in postoperative patients and to enable the early detection of potential complications.^1^ In the PACU, early detection and proper management of postoperative complications are essential for improving clinical outcomes and optimising perioperative care.^2^

The increasing volume of surgical procedures has led to a higher proportion of high-risk patients who require advanced monitoring in the postoperative period. Consequently, the efficient use of limited intensive care resources has become increasingly important. In this context, high-dependency units (HDUs), also referred to as intermediate or step-down care units, play a critical role between general wards and intensive care units in many healthcare systems.^3^

At our centre, to address this need, a PACU with characteristics consistent with those of an HDU, as described in the literature, is available. This unit admits patients identified as high risk during preoperative assessment and allows for close postoperative haemodynamic monitoring, short- to medium-term mechanical ventilation, inotropic support, and advanced monitoring.

In recent years, the surgical stress response has been shown to influence the clinical course through systemic inflammatory and metabolic changes. Accordingly, ratios and indices derived from routine laboratory parameters have attracted increasing interest in postoperative risk assessment.^4, 5, 6, 7, 8^ The ease of calculation and low cost of these indicators provide practical advantages for their use in the early postoperative period, particularly in time-critical settings such as the PACU. Although studies have investigated risk factors for adverse short-term clinical outcomes in postoperative intensive care unit and PACU populations, data specifically evaluating these risks in PACU populations with HDU characteristics remain limited.

Accordingly, the present study sought to investigate the relationship between systemic inflammatory and metabolic indices and short-term adverse clinical outcomes and to determine their predictive performance among adults receiving care in the PACU.

## Methods

### Study Design and Ethical Approval

The study protocol was approved by the Scientific Research Ethics Committee of University of Health Sciences Türkiye, Kocaeli City Hospital (approval no.: 2025-181, date: 08.01.2026). Owing to the retrospective design, the requirement for written informed consent was waived. All procedures were carried out in accordance with the principles of the Declaration of Helsinki. This single-center, retrospective observational study was prepared in line with the STROBE guidelines.^9^

### Study Population

This study was conducted at University of Health Sciences Türkiye, Kocaeli City Hospital, a tertiary care teaching and research hospital located in Kocaeli, Türkiye. Adults (≥18 years) admitted to the PACU after surgical procedures between 1 March 2025 and 30 June 2025 were retrospectively identified through the hospital’s electronic records and were enrolled in the study.

Patients who were admitted to the PACU more than once for repeat postoperative surgical procedures, as well as individuals younger than 18 years, were excluded.

### Data Collection

The data analysed in this study were obtained retrospectively from the hospital’s electronic medical record system. The collected data were evaluated across five main categories: demographic characteristics, clinical and surgical variables, laboratory findings, inflammatory and metabolic indices, and postoperative outcomes. Demographic data included age, sex, American Society of Anesthesiologists (ASA) physical status score, blood group, and comorbidities, including cardiovascular, pulmonary, renal, neurological, and endocrine-metabolic diseases, malignancy, and other conditions. Clinical and surgical variables included the following: surgical specialty performing the operation; urgency of surgery (elective or emergency); duration of surgery; type of anaesthesia administered; intraoperative use of erythrocyte suspension (ES), fresh frozen plasma (FFP), and colloids; airway status on arrival at the PACU (extubated, intubated, or tracheostomised); and the need for inotropic support at PACU admission.

Laboratory parameters were obtained from blood samples collected at the time of the first admission to the PACU. Arterial blood gas analysis included the following parameters: pH, partial pressure of carbon dioxide (PCO_2_, mmHg), partial pressure of oxygen (PO_2_, mmHg), oxygen saturation (SO_2_, %), lactate (mmol L^-1^), bicarbonate (HCO_3_⁻, mmol L^-1^), and base excess (mmol L^-1^).

Haematological parameters included platelet count (PLT, ×10³/µL), mean platelet volume (MPV, fL), plateletcrit (%), platelet distribution width (PDW, %), white blood cell count (WBC, ×10³/µL), haemoglobin (Hb, g dL^-^¹), haematocrit (Htc, %), mean corpuscular haemoglobin (MCH, pg), mean corpuscular volume (MCV, fL), mean corpuscular haemoglobin concentration (MCHC, g dL^-^¹), and absolute neutrophil, lymphocyte, monocyte, eosinophil, and basophil counts (×10³/µL).

Biochemical parameters included glucose (mg dL^-^¹), creatinine (mg dL^-^¹), estimated glomerular filtration rate (eGFR, mL min^-^¹ 1.73 m²), blood urea nitrogen (BUN, mg dL^-^¹), C-reactive protein (CRP, mg L^-^¹), aspartate aminotransferase (AST, U L^-^¹), alanine aminotransferase (ALT, U L^-^¹), lactate dehydrogenase (LDH, U L^-^¹), albumin (g dL^-^¹), total protein (g dL^-^¹), total bilirubin (mg dL^-^¹), direct bilirubin (mg dL^-^¹), calcium (mg dL^-^¹), magnesium (mg dL^-^¹), sodium (mmol L^-^¹), potassium (mmol L^-^¹), and amylase (U L^-^¹).

Coagulation parameters included prothrombin time (PT, second), activated partial thromboplastin time (aPTT, second), and international normalised ratio (INR).

Inflammatory and metabolic indices were calculated from laboratory data obtained at first admission to the PACU. These indices included: the monocyte-to-lymphocyte ratio (MLR) (MLR=monocytes/lymphocytes), PLT-to-lymphocyte ratio [platelet-to-lymphocyte ratio (PLR)=PLTs/lymphocytes], neutrophil-to-lymphocyte ratio (NLR) (NLR=neutrophils/lymphocytes), lymphocyte-to-monocyte ratio (LMR) (LMR=lymphocytes/monocytes), neutrophil-lymphocyte-PLT ratio (NLPR) [NLPR=(neutrophils/lymphocytes) ×PLTs], mean PLT volume-to-PLT ratio (MPR) (MPR=MPV/PLTs), systemic immune-inflammation index [SII=(neutrophils×PLTs]/lymphocytes), pan-immune-inflammation value [pan-immune-inflammation value=(neutrophils×monocytes×PLTs)/lymphocytes], CRP-to-lymphocyte ratio (CLR) (CLR=CRP/lymphocytes), CRP-to-albumin ratio (CAR) (CAR=CRP/albumin), neutrophil-to-albumin ratio (NAR) (NAR=neutrophils/albumin), albumin-to-creatinine ratio (ACR=albumin/creatinine), and BUN-to-albumin ratio (BAR) (BAR=BUN/albumin).

### Bias and Sample Size Considerations

Because of the retrospective design, the study may be subject to selection and information bias. To reduce this potential bias, all consecutive patients meeting the predefined eligibility criteria were enrolled. Cases with missing data were not excluded; instead, missing values were handled using multiple imputation and included in the analysis, with the aim of reducing bias related to missing data.^10^

An a priori sample size calculation was not conducted because of the study’s retrospective design. The study population included all patients who fulfilled the eligibility criteria during the predefined time frame.

### Definition of Clinical Outcomes

In routine PACU practice, patients were initially monitored during the early postoperative period and subsequently transferred to the general ward or an advanced intensive care unit, according to their clinical status. Taking into account both the routine clinical workflow of the PACU and the recommendations of current post-anaesthesia care guidelines, patients who remained in the PACU for ≥24 hours were classified as having a prolonged PACU stay.^1^

Accordingly, in this study, a PACU length of stay of ≥24 hours was defined as a prolonged PACU stay. Clinical outcomes were classified as binary variables. Patients with a prolonged PACU stay, those transferred to an advanced intensive care unit, and those who died within the first 7 postoperative days were classified as having a poor short-term clinical outcome. In contrast, the good short-term clinical outcome group consisted of patients without a prolonged PACU stay, patients discharged directly to the general ward, and patients who did not experience death within the first 7 postoperative days.

### Statistical Analysis

Statistical analyses were performed using IBM SPSS Statistics for Windows, version 27.0 (IBM Corp., Armonk, NY, USA). Descriptive statistics were presented as mean ± standard deviation, median (minimum-maximum), or frequency and percentage (n, %). The distribution of variables was assessed using the Kolmogorov-Smirnov test. Independent continuous variables with a normal distribution were analysed using the Student’s t test and reported as mean ± standard deviation, whereas those without normal distribution were analysed using the Mann-Whitney U test and reported as median (minimum-maximum). Independent categorical variables were analysed using the Pearson chi-square test (with Yates’ continuity correction when appropriate) or Fisher’s exact test, as appropriate. Categorical data were presented as frequencies and percentages. A *P* value of <0.05 was considered statistically significant.

Missing data were initially evaluated using missing- data analysis. Little’s missing completely at random test was applied to assess the randomness of missing values for comorbidities, pH, pCO_2_, pO_2_, SO_2_, HCO_3_^-^, base excess (mmol L^-^¹), lactate, MPV, plateletcrit, PDW, AST, ALT, LDH, potassium, direct bilirubin, aPTT, duration of surgery, ES, FFP, and colloid replacement. The test result (*P*=0.763) confirmed that the data were missing completely at random. Multiple imputation (m=5) was applied to these variables.

Primary statistical analyses were performed using the observed (original) dataset. Multiple imputation analysis was conducted as a sensitivity analysis to evaluate the potential impact of missing data on the results. Post-imputation results were pooled according to Rubin’s rules, and only findings identified as sensitive to imputation were explicitly reported in the text.

Inflammatory and metabolic indices identified with* P *< 0.20 in univariable analyses^11, 12^ were evaluated for multicollinearity using the variance inflation factor (VIF). Indices with a VIF value >5 [NLR and natural logarithm of NLR multiplied by PLT count (lnNLRP)] were excluded from the multiple logistic regression model because of multicollinearity. The remaining indices [PLR, MLR, CAR, CLR, BAR, ACR, and ln(MPR)] were included in the multivariable model.

During univariable analyses, NLPR and MPR demonstrated overdispersion and wide confidence intervals (CIs). Therefore, to reduce the influence of extreme values and improve model stability, these indices were subjected to natural logarithmic transformation and evaluated as ln(NLPR) and ln(MPR) in regression analyses.

In addition to the indices, variables with *P* < 0.20 in univariable analyses—age, ASA score, inotropic use on arrival, administration of ES and FFP, and airway status on arrival to the PACU—were included in the model as potential confounders. The final model was obtained using the backward likelihood ratio approach to achieve a more parsimonious structure. In this final model, inotropic use on arrival to the PACU and the MLR and BAR indices remained independently significant.

To assess the stability of the final multiple model and to evaluate potential residual multicollinearity, ridge regression was performed as a supplementary analysis using the same independent variables in NCSS statistical software, version 11 (NCSS, LLC, Kaysville, Utah, USA). Ridge regression analysis (k=0.005) demonstrated that the model was statistically significant (F=4.033;* P* < 0.001) and that multicollinearity was effectively controlled.

Receiver operating characteristic (ROC) analysis was conducted to examine the discriminative capacity of MLR and BAR, which were found to be independently related to poor short-term clinical outcomes. The area under the curve (AUC) was estimated together with its 95% CI. Optimal cut-off values were identified at the point where the difference between sensitivity and specificity was smallest. For these cut-off points, sensitivity, specificity, positive predictive value, negative predictive value, and overall accuracy were also calculated.

Because the primary outcome was defined as a composite endpoint including prolonged PACU stay, transfer to the intensive care unit, and 7-day mortality, additional sensitivity analyses were performed by evaluating the individual components of this composite outcome separately. In this context, prolonged PACU stay, transfer to the intensive care unit, and 7-day mortality were each analyzed using multivariable logistic regression models.

Furthermore, ROC analysis was conducted to evaluate the ability of the BAR to predict 7-day mortality. The optimal cut-off value was determined based on the point at which the difference between sensitivity and specificity was minimized. In this analysis, sensitivity, specificity, positive predictive value, negative predictive value, and overall accuracy were calculated, together with their corresponding 95% CIs.

## Results

### Participants and Flow Diagram

**Figure 1:**
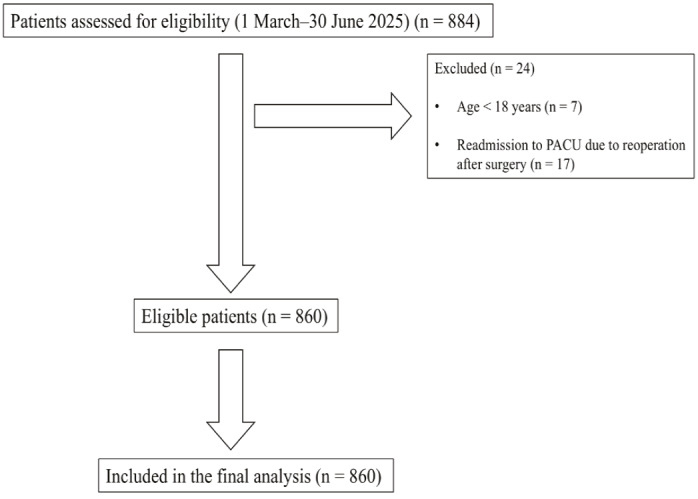
Flow chart. PACU, post-anaesthesia care unit.

Of the 884 patients screened for eligibility, those younger than 18 years (n = 7) and those readmitted to the PACU for repeat surgical procedures in the postoperative period (n = 17) were excluded. After applying the exclusion criteria, 860 patients were included in the final analysis (Figure 1).

### Baseline Characteristics

Among the 860 patients included in the study, the median age was 68 years (range, 18-98). No statistically significant differences in age or sex distribution were observed between patients with and without poor short-term clinical outcomes (all *P* > 0.05).

At least one comorbidity was present in 90.2% of the patients. No significant differences were observed between the groups regarding the presence of overall comorbidity or specific comorbid conditions, including cardiovascular, pulmonary, malignant, neurological, endocrine-metabolic, and other diseases (all *P* > 0.05). In contrast, the prevalence of renal disease was significantly higher in the poor short-term clinical outcome group (*P*=0.002).

No significant differences between groups were observed in blood group or surgical specialty distribution (*P* > 0.05). However, ASA scores differed significantly between the groups (*P* < 0.001).

Among the baseline characteristics presented in Table 1, missing data on comorbidities were identified for 23 patients. Variables related to comorbidities were therefore considered sensitive to the missing data structure.

### Perioperative and Postoperative Characteristics

No statistically significant differences were observed between groups in surgical urgency, anaesthesia type, duration of surgery, or colloid use (all *P* > 0.05).

In contrast, airway status at PACU admission differed significantly between the groups, with a higher proportion of patients in the poor short-term clinical outcome group admitted while intubated (*P*=0.002). Inotropic use was also significantly more frequent in this group (*P* < 0.001). Additionally, ES and FFP replacements were significantly higher in the poor short-term clinical outcome group (*P*=0.023 and *P*=0.014, respectively).

Missing data were present for duration of surgery (min) (n = 1), ES replacement (n = 8), FFP replacement (n = 8), and colloid use (n = 8). Multiple imputation analyses applied to these variables did not identify any inconsistent parameters that would indicate sensitivity to the missing-data structure. Perioperative and postoperative characteristics are summarised in Table 2.

### Laboratory Findings

Among blood gas parameters, PCO_2_ levels differed significantly between the groups (*P*=0.026). Hb and Htc values differed significantly between the groups (*P*=0.035 and *P*=0.029, respectively).

When leukocyte subgroups were examined, lymphocyte and eosinophil counts differed significantly between the groups (*P*=0.009 and *P*=0.049, respectively), whereas no significant differences were observed for the other leukocyte subgroups (*P* > 0.05).

Among renal function parameters, levels of creatinine, GFR, and BUN differed significantly between the groups (*P*=0.004, *P*=0.004, and *P* < 0.001, respectively). In addition, CRP and albumin levels differed significantly between the groups (*P*=0.044 and *P*=0.031, respectively).

Among coagulation parameters, PT and INR values differed significantly between the groups (*P*=0.016 and *P* < 0.001, respectively), whereas no significant differences were observed in the remaining coagulation parameters (*P* > 0.05).

Missing data were present for the following variables: pH, PCO_2_, HCO_3_, and base excess (63 patients); PO_2 _(80 patients); SO_2_ (73 patients); lactate (70 patients); MPV, plateletcrit, and PDW (14 patients); AST (14 patients); ALT (22 patients); LDH (8 patients); direct bilirubin (6 patients); potassium (5 patients); and aPTT (38 patients). In the multiple imputation assessment, the findings for PCO_2_, base excess, and aPTT were identified as sensitive to the structure of the missing data. All laboratory findings are summarised in Table 3.

### Systemic Inflammatory and Metabolic Indices

PLR and MLR values differed significantly between the groups (*P*=0.033 and *P*=0.028, respectively). CAR and CLR were significantly higher in the poor short-term clinical outcome group (*P*=0.033 and *P*=0.022, respectively). Similarly, LMR values differed significantly between the groups (*P*=0.033).

Among metabolic indices, BAR was markedly higher in the poor short-term clinical outcome group, and this difference was statistically significant (*P* < 0.001). A significant difference in ACR was observed between the groups (*P*=0.001).

No statistically significant differences were observed between the groups with respect to NLR, SII, PIV, MPR, NLPR, or NAR (all *P* > 0.05). Systemic inflammatory and metabolic indices are summarised in Table 3.

### Risk Factors for Poor Short-term Clinical Outcomes

The results of the univariable and multiple analyses are presented in Table 4.

In univariable analysis, an ASA IV score was associated with a significantly increased risk of poor short-term clinical outcomes [Odds ratio (OR)=4.111, 95% CI: 1.074-15.737; *P*=0.039]. Similarly, the use of inotropic agents on arrival at the PACU was associated with a marked increase in risk of poor short-term clinical outcomes (OR=4.398, 95% CI: 2.150-8.996; *P* < 0.001).

Perioperative administration of ES (OR=1.606, 95% CI: 1.066-2.420; *P*=0.024) and perioperative administration of FFP (OR=2.047, 95% CI: 1.147-3.655; *P*=0.015) were also identified as factors associated with an increased risk of poor short-term clinical outcomes, and the findings for these two parameters were not sensitive to the missing data structure.

Among patients admitted to the PACU while intubated, the risk of poor short-term clinical outcomes was significantly higher than in extubated patients (OR=2.061, 95% CI: 1.379-3.081; *P* < 0.001).

When laboratory parameters were evaluated, higher Hb (OR=0.913, 95% CI: 0.847-0.984; *P*=0.017) and Htc (OR=0.972, 95% CI: 0.948-0.997; *P*=0.029) levels were associated with a reduced risk of poor short-term clinical outcomes, indicating a protective effect. In contrast, increased levels of creatinine (OR=1.665, 95% CI: 1.237-2.242; *P*=0.001), BUN (OR=1.036, 95% CI: 1.021-1.052; *P* < 0.001), and CRP (OR=1.005, 95% CI: 1.002-1.008; *P*=0.001) were associated with an increased risk of poor short-term clinical outcomes.

GFR (OR=0.992, 95% CI: 0.987-0.997; *P*=0.001), albumin (OR=0.719, 95% CI: 0.576-0.899; *P*=0.004), and total protein (OR=0.861, 95% CI: 0.748-0.991; *P*=0.037) levels were protective against poor short-term clinical outcomes. Calcium levels similarly showed a significant protective association (OR=0.808, 95% CI: 0.659-0.990; *P*=0.040).

Among coagulation parameters, increases in aPTT (OR=1.024, 95% CI: 1.000-1.049; *P*=0.046), PT (OR=1.198, 95% CI: 1.076-1.334; *P*=0.001), and, particularly INR (OR=8.938, 95% CI: 3.873-20.631; *P* < 0.001) were associated with markedly increased risk of poor short-term clinical outcomes. The findings for aPTT were sensitive to the structure of missing data.

When systemic inflammatory and metabolic indices were examined, increases in NLR, PLR, MLR, CAR, CLR, BAR, and lnNLPR were associated with an increased risk of poor short-term clinical outcomes (all *P* < 0.05). In contrast, increasing ACR values were associated with a reduced risk of poor short-term clinical outcomes, indicating a protective effect (OR=0.896, 95% CI: 0.833-0.963; *P*=0.003).

In multiple logistic regression analysis, after adjustment for potential confounding variables, the use of inotropic agents on arrival at the PACU remained an independent risk factor for poor short-term clinical outcomes (OR=2.785, 95% CI: 1.320-5.875; *P*=0.007).

Among systemic inflammatory and metabolic indices, an increase in MLR was independently associated with an increased risk of poor short-term clinical outcomes (OR=1.668, 95% CI: 1.118-2.488; *P*=0.002). Similarly, higher BAR values were independently associated with poor short-term clinical outcomes (OR=1.100, 95% CI: 1.052-1.150; *P* < 0.001).

To validate the stability of the multiple logistic regression model and to assess potential residual multicollinearity, ridge regression analysis was performed using the same independent variables. In ridge regression analysis, VIF values for all variables included in the model ranged from 1.19 to 3.57. Examination of standardised regression coefficients showed that BAR had the strongest effect on the dependent variable (β=0.107), followed by ASA score (β=0.078), MLR (β=0.075), and inotropic use on arrival at the PACU (β=0.067). All of these variables demonstrated a positive effect on the dependent variable. In addition, the direction and relative magnitude of the coefficients were consistent with those obtained from the logistic regression model (Supplementary Table 1).

### Sensitivity Analyses of Individual Outcome Components

Because the composite outcome consisted of components with different clinical weights, additional sensitivity analyses were performed. In the multivariable analysis conducted for prolonged PACU stay, the BAR (OR=1.097, 95% CI: 1.058-1.137; *P* < 0.001), the MLR (OR=1.822, 95% CI: 1.266-2.622; *P*=0.001), and the requirement for inotropic support at PACU admission (OR=3.489, 95% CI: 1.967-6.188; *P* < 0.001) were independently associated with the outcome (Supplementary Table 2).

In the analysis of 7-day mortality, BAR (OR=1.121, 95% CI: 1.063-1.183; *P *< 0.001) and the need for inotropic support (OR=17.2, 95% CI: 7.798-37.935; *P* < 0.001) remained significant, whereas MLR was not statistically significant (*P*=0.645) (Supplementary Table 3). In the intensive care unit (ICU) transfer analysis, MLR was independently associated with the outcome (OR=1.471, 95% CI: 1.043-2.075; *P*=0.028), whereas BAR was not statistically significant (*P*=0.645) (Supplementary Table 4).

Additionally, an exploratory ROC analysis was performed to evaluate BAR’s ability to predict 7-day mortality (Supplementary Figure 1, Supplementary Table 5). In this analysis, BAR demonstrated good discriminative performance for predicting mortality (AUC=0.850, 95% CI: 0.780-0.919; *P* < 0.001). The optimal cut-off value was determined as ≥6.715. At this threshold, sensitivity was 75.6% (95% CI: 59.7-87.6), specificity was 76.0% (95% CI: 73-79), positive predictive value was 13.7% (95% CI: 11.3-16.4), negative predictive value was 98.4% (95% CI: 97.3-99.1), and overall accuracy was 76.1% (95% CI: 73.1-78.9).

### ROC Analysis of MLR for Predicting Poor Short-term Clinical Outcomes

**Figure 2:**
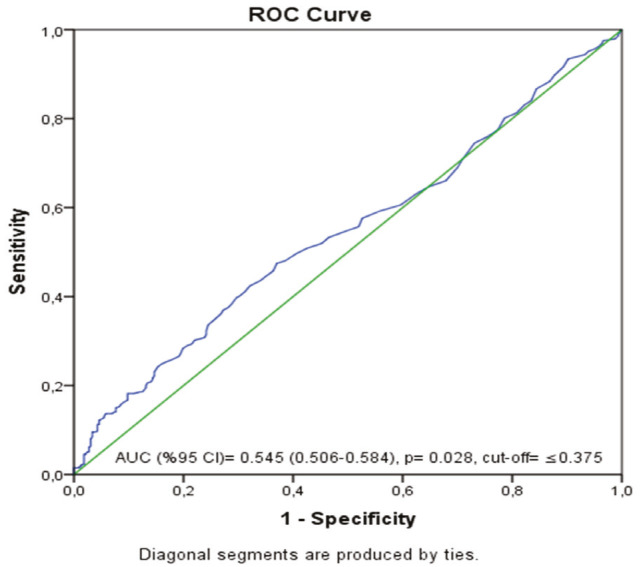
ROC curve of the monocyte-to-lymphocyte ratio for predicting short-term poor clinical outcomes. ROC, receiver operating characteristic; AUC, area under the curve; CI, confidence interval.

ROC analysis of MLR showed an AUC of 0.545 for  predicting poor short-term clinical outcomes (95% CI: 0.506-0.584; *P*=0.028) (Figure 2). The optimal cut-off value, defined as the point with the smallest difference between sensitivity and specificity, was ≥0.375. At this cut-off value, sensitivity was 53.3% (95% CI: 49-57.6) and specificity was 53.5% (95% CI: 48-59). The positive predictive value was 65.1% (95% CI: 61.9-68.3), the negative predictive value was 41.3% (95% CI: 38-44.6), and the overall accuracy was 53.4% (95% CI: 50-56.8) (Table 5).

### ROC Analysis of BAR for Predicting Poor Short-term Clinical Outcomes

**Figure 3:**
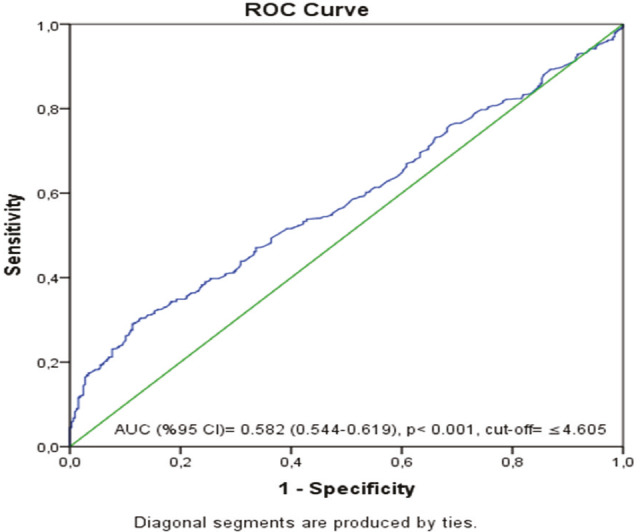
ROC curve of the blood urea nitrogen-to-albumin ratio for predicting short-term poor clinical outcomes. ROC, receiver operating characteristic; AUC, area under the curve; CI, confidence interval.

ROC analysis of BAR yielded an AUC of 0.582 for predicting poor short-term clinical outcomes (95% CI: 0.544-0.619; *P* < 0.001) (Figure 3). The optimal cut-off value, defined using the same criterion, was ≥4.605. At this cut-off value, sensitivity was 54.4% (95% CI: 50.1-58.7) and specificity was 54.1% (95% CI: 48.6-59.6). The positive predictive value was 65.9% (95% CI: 62.7-69); the negative predictive value was 42.1% (95% CI: 38.9-45.5); and the overall accuracy was 54.3% (95% CI: 50.9-57.7) (Table 5).

## Discussion

This retrospective observational study investigated the potential association between systemic inflammatory and metabolic indices and short-term adverse clinical outcomes in adult patients admitted to the PACU. In multiple logistic regression analysis, after adjustment for potential clinical and biochemical confounders, MLR and BAR were associated with short-term adverse clinical outcomes.

However, the AUC values for both indices in ROC analyses were close to 0.5, indicating that these parameters have limited discriminative ability in predicting clinical outcomes. Although the findings suggest a statistical association of BAR and MLR with adverse clinical outcomes, they do not indicate that these indices can be used as strong predictors on their own. In addition, this study did not evaluate whether adding these indices to standard clinical risk models, including the ASA physical status classification or other perioperative clinical variables, would improve predictive performance. Accordingly, when considering the clinical use of these indices, it should be noted that the findings may primarily reflect a biological association and that their predictive performance appears limited.

Because the primary outcome in our study was defined as a composite including prolonged PACU stay, ICU transfer, and 7-day mortality, additional sensitivity analyses were performed to determine whether the findings differed according to the individual components. In these analyses, the multivariable analysis for prolonged PACU stay showed that BAR, MLR, and the requirement for inotropic agents at PACU admission remained significant. In contrast, in the mortality analysis, only BAR and the requirement for inotropic agents remained significant, whereas MLR lost statistical significance. In the ICU transfer analysis, MLR was independently associated with the outcome, whereas BAR was not significant. These findings suggest that inflammatory and metabolic indices may reflect different aspects of postoperative clinical deterioration. In the additional ROC analysis for mortality, the discriminative performance of BAR was markedly higher (AUC=0.850, 95% CI: 0.780-0.919). Moreover, the high negative predictive value (98.4%) suggests that low BAR levels may be useful in identifying patients at low risk of short-term mortality. This observation suggests that composite outcomes that combine results with different clinical weights may attenuate the predictive performance of certain biomarkers.

Surgical procedures may affect host defence mechanisms through local tissue injury and disruption of physiological barriers; this process can increase susceptibility to infectious agents and trigger both local and systemic inflammatory responses.^4^ The magnitude and duration of the inflammatory response may vary depending on the type of surgical procedure, the extent of surgical trauma, and patient-related clinical characteristics.^4^ During the postoperative period, this inflammatory response is modulated by the dynamic interplay between pro-inflammatory and anti-inflammatory components of the immune system.^4^ Disruption of this balance may lead to uncontrolled systemic inflammation, potentially predisposing patients to organ dysfunction and being associated with increased morbidity and mortality.^4^

In recent years, inflammatory markers have gained attention as potential prognostic indicators of mortality and adverse clinical outcomes in postoperative patients.^13^ Incorporation of these markers into risk assessment processes has been reported to contribute to the development of personalised treatment strategies and to improvement in postoperative patient outcomes.^13^ The existing literature comprises studies evaluating various inflammatory and metabolic markers across different surgical procedures; however, these studies exhibit considerable heterogeneity in terms of patient populations and surgical characteristics.^14, 15, 16^ Indeed, Kilinc et al.^13^ reported that postoperative inflammation and nutritional condition were linked to mortality in patients undergoing surgery for femoral fracture.

In this study, the association of inflammatory and metabolic markers with poor short-term clinical outcomes was evaluated in adult patients from a heterogeneous surgical population monitored in the PACU. In contrast to most studies, which primarily focus on specific surgical subgroups, the inclusion of patients requiring PACU monitoring after diverse surgical procedures allows for assessment of the potential utility of these indices in real-world clinical practice. The finding that BAR and MLR remained associated with poor clinical outcomes after adjustment for other variables reflecting clinical severity in multiple analyses suggests that these indices may contribute to risk stratification within the PACU population.

BAR is an index that reflects the combined effects of multiple pathophysiological processes, including renal perfusion and clearance, hepatic synthetic capacity, nutritional status, and endothelial integrity.^17^ Because BUN reflects renal function and metabolic status, while albumin plays a critical role in maintaining fluid balance and oncotic pressure, the combined assessment of these two parameters has been emphasised for its clinical relevance.^17, 18^ Evidence from earlier studies suggests a relationship between BAR and mortality or morbidity in different patient groups.^15, 17, 19, 20, 21, 22^ In a study by Chen et al.^15^, BAR was linked to long-term mortality in patients treated surgically for hip fracture and was proposed as a practical indicator for early risk evaluation. However, differences between the patient populations and outcome measures reported in the literature and those in the present study limit direct comparisons of findings regarding the prognostic value of BAR. Heterogeneity in patient populations and clinical contexts may also contribute to variability in the discriminative performance reported in ROC analyses. This observation suggests that, rather than functioning as a strong standalone predictor, BAR may be more appropriately considered as a complementary marker within multiparametric risk assessment models.

In the present study, MLR was found to be independently associated with poor short-term clinical outcomes in adult patients monitored in the PACU. As a marker of systemic immune response, the prognostic value of MLR has been explored in various clinical settings and has been linked to adverse outcomes, particularly among patients with malignancies and surgical populations.^23, 24, 25^ In addition, studies involving elderly patients with hip fractures have reported associations between MLR and both mortality and intensive care unit admission.^14, 16, 26^ However, the moderate discriminative performance of MLR observed in our ROC analysis suggests that, rather than serving as a strong standalone predictor, this index—similar to BAR—may be more appropriately considered as a complementary marker within multiparametric risk assessment models.

In this study, the use of inotropic agents upon admission to the PACU was independently associated with poor short-term clinical outcomes. This finding suggests that haemodynamic instability in the early postoperative period may indicate a more complicated clinical trajectory and a greater need for close monitoring. The potential for haemodynamic instability to increase the risk of organ dysfunction and the need for higher levels of care may plausibly explain the observed association between inotropic support on PACU admission and poor short-term clinical outcomes.

### Study Limitations

This study has several limitations. Owing to its retrospective and single-center design, causal relationships cannot be established, and the generalisability of the findings to other centres is limited. In addition, the heterogeneous nature of the study population—encompassing multiple surgical specialties, variable anaesthetic techniques, and a broad spectrum of comorbidities—may have complicated the interpretation of the associations between inflammatory and metabolic indices and poor short-term clinical outcomes.

The use of electronic medical records as the primary data source may have precluded detailed evaluation of certain potential confounding variables, such as analgesic regimens, sedative dosages, neurological assessments, and preoperative anticoagulant therapy. Furthermore, as laboratory parameters reflect only the initial postoperative values obtained at PACU admission, it remains unclear to what extent these measurements distinguish chronic physiological status from the acute surgical stress response. Outcome assessment was also limited to the early postoperative period, and data on longer-term outcomes, including 30-day mortality, were not available. Accordingly, the findings should be interpreted as reflecting short-term clinical outcomes.

Another limitation is the use of a composite primary outcome that combines prolonged PACU stay, ICU transfer, and 7-day mortality. As these components differ in their clinical significance, the use of a composite endpoint may have influenced the observed predictive performance of the investigated indices. Although additional sensitivity analyses examining individual outcome components were performed, future studies may benefit from evaluating these outcomes separately.

Decisions regarding PACU admission and escalation to higher levels of care during the postoperative period were partly based on clinicians’ judgement, suggesting that unmeasured clinical factors may have influenced outcomes. Despite the use of multiple imputation to handle missing data, the potential impact of this approach on the results cannot be entirely excluded. Finally, our institution’s PACU has characteristics similar to those of an HDU, which may restrict the generalisability of these findings to PACU settings intended primarily for brief postoperative recovery.

## Conclusion

In this retrospective observational study, metabolic and inflammatory indices derived from routine laboratory data were evaluated among adult patients admitted to the PACU; BAR and MLR were associated with short-term adverse clinical outcomes. However, the AUC values obtained in the ROC analyses were close to 0.5, indicating that the discriminative performance of these indices is limited. Although a statistical association was observed between these indices and adverse clinical outcomes, these findings do not support the use of these parameters as strong predictive tools on their own. These indices may instead reflect certain aspects of postoperative physiological stress and should be interpreted cautiously within the broader clinical context. Further prospective, multicentre studies are needed to clarify the potential role of metabolic and inflammatory indices in postoperative risk assessment.

## Ethics

**Ethics Committee Approval:** The study protocol was approved by the Scientific Research Ethics Committee of University of Health Sciences Türkiye, Kocaeli City Hospital (approval no.: 2025-181, date: 08.01.2026).

**Informed Consent:** Owing to the retrospective design, the requirement for written informed consent was waived.

## Supplementary Materials

Supplementary Figure 1 https://d2v96fxpocvxx.cloudfront.net/beb8919b-f013-4ea1-b1c8-40332e840fe1/content-images/fcaef28c-1cd3-45cd-a935-f16098c4b88f.pdf

Supplementary Tables 1-5 https://d2v96fxpocvxx.cloudfront.net/beb8919b-f013-4ea1-b1c8-40332e840fe1/content-images/ba0a05d8-be93-427b-bc9e-eb766e80b7c1.pdf Supplementary Figure 1: https://

## Figures and Tables

**Table 1. Baseline Characteristics of the Study Population According to Short-term Clinical Outcomes:** 

**Variables**	**All patients** **(n = 860)**	**Good outcome (n = 327)**	**Poor outcome (n = 533)**	*P value
Age (years)	68 (18-98)	67 (21-92)	69 (18-98)	0.204^m^
**Sex**				
Female	388 (45.1)	144 (44)	244 (45.8)	0.618^x^
Male	472 (54.9)	183 (56)	289 (54.2)	
Comorbidities	755 (90.2) [23]	274 (89) [19]	481 (90.2) [4]	0.356^x^^ꝭ^
Cardiovascular diseases	539 (64.4) [23]	293 (65.9) [19]	336 (63.5) [4]	0.486^x^
Neurological diseases	102 (12.2) [23]	29 (9.4) [19]	73 (13.8) [4]	0.062^x^^ꝭ^
Pulmonary diseases	146 (17.4) [23]	50 (16.2) [19]	96 (18.1) [4]	0.482^x^
Endocrine and metabolic disorders	325 (38.8) [23]	117 (38) [19]	208 (39.3) [4]	0.703^x^
Renal diseases	62 (7.4) [23]	11 (3.6) [19]	51 (9.6) [4]	0.002*^y^ **^ꝭ^**
Malignancy	142 (17) [23]	44 (14.3) [19]	98 (18.5) [4]	0.115^x^
Other comorbidities	65 (7.8) [23]	24 (7.8) [19]	41 (7.8) [4]	1.000^y^^ꝭ^
**Blood types**				
O Rh^-^	44 (5.1)	18 (5.5)	26 (4.9)	0.896^x^
O Rh^+^	233 (27.1)	87 (26.6)	146 (27.4)	
A Rh^-^	58 (6.7)	22 (6.7)	36 (6.8)	
A Rh^+^	350 (40.7)	129 (39.4)	221 (41.5)	
B Rh^-^	14 (1.6)	4 (1.2)	10 (1.9)	
B Rh^+^	116 (13.5)	46 (14.1)	70 (13.1)	
AB Rh^-^	7 (0.8)	4 (1.2)	3 (0.6)	
AB Rh^+^	38 (4.4)	17 (5.2)	21 (3.9)	
**ASA physical Status**				
ASA I	10 (1.2)	5 (1.5)	5 (0.9)	<0.001*^z^
ASA II	204 (23.7)	90 (27.5)	114 (21.4)	
ASA III	544 (63.3)	213 (65.1)	331 (62.1)	
ASA IV	92 (10.7)	18 (5.5)	74 (13.9)	
ASA V	10 (1.2)	1 (0.3)	9 (1.7)	
**Surgical specialties**				
Neurosurgery	97 (11.3)	37 (11.3)	60 (11.3)	0.207^x^
General surgery	350 (40.7)	118 (36.1)	232 (43.5)	
Thoracic surgery	47 (5.5)	20 (6.1)	27 (5.1)	
Obstetrics and gynecology	54 (6.3)	21 (6.4)	33 (6.2)	
Orthopedics	148 (17.2)	55 (16.8)	93 (17.4)	
Urology	106 (12.3)	51 (15.6)	55 (10.3)	
Other specialties	58 (6.7)	25 (7.6)	33 (6.2)	

Data are presented as number (%) or median (minimum-maximum). ^x^: Pearson’s chi-squared test; ^y^: Pearson’s chi-squared test with Yates’ continuity correction; ^z^: Fisher’s exact test; ^m^: Mann-Whitney U test, ^ꝭ^: variable sensitive to missing data. *: *P* value <0.05 was considered statistically significant; ASA, American Society of Anesthesiologists

**Table 2. Perioperative and Postoperative Characteristics of the Study Population According to Short-term Clinical Outcomes:** 

**Variables**	**All patients** **(n = 860)**	**Good outcome** **(n = 327)**	**Poor outcome** **(n = 533)**	*P value
**Surgical Urgency**				
Elective	612 (71.2)	239 (73.1)	373 (70)	0.329^x^
Emergency	248 (28.8)	88 (26.9)	160 (30)	
**Type of anaesthesia**				
General anaesthesia (TIVA)	17 (2)	9 (2.8)	8 (1.5)	0.624^z^
General anaesthesia (inhalational)	778 (90.5)	293 (89.6)	485 (91)	
Regional anaesthesia	57 (6.6)	22 (6.7)	35 (6.6)	
Sedoanalgesia	8 (0.9)	3 (0.9)	5 (0.9)	
**Airway status on PACU admission**				
Extubated	690 (80.2)	281 (85.9)	409 (76.7)	0.002*^x^
Intubated	148 (17.2)	37 (11.3)	111 (20.8)	
Tracheostomized	22 (2.6)	9 (2.8)	13 (2.4)	
Inotrope requirement at PACU admission	68 (7.9)	9 (2.8)	59 (11.1)	<0.001*^x^
Duration of surgery (minute)	120 (15-630) [11]	120 (20-570) [5]	120 (15-630) [6]	0.322^m^^¥^
Erythrocyte suspension transfusion	128 (15) [8]	37 (11.5) [4]	91 (17.2) [4]	0.023*^x^ **^¥^**
Fresh frozen plasma transfusion	67 (7.9) [8]	16 (5) [4]	51 (9.6) [4]	0.014*^x^ **^¥^**
Colloid administration	117 (13.9) [18]	48 (15.2) [12]	69 (13.1) [6]	0.384^x^^¥^
**Discharge destination**				
Ward	796 (92.6)	327 (100)	469 (88)	N/A
Level 2-3 ICU	37 (4.3)	0 (0)	37 (6.9)	
**Mortality**				
Death in the PACU	27 (3.1)	0 (0)	27 (5.1)	N/A
7 day mortality	41 (4.8)	0 (0)	41 (7.7)	N/A

Data are presented as number (%) or median (minimum-maximum). Missing data are indicated as [n], ^x^: Pearson’s chi-squared test; ^z^: Fisher’s exact test; ^m^: Mann-Whitney U test, *: *P* value <0.05 was considered statistically significant. ^¥^: Not sensitive to missing data, N/A, not applicable; ICU, intensive care unit; TIVA, total intravenous anaesthesia; PACU, post-anaesthesia care unit

**Table 3. Laboratory Findings and Systemic Inflammatory and Metabolic Indices According to Short-term Clinical Outcomes:** 

**Variables**	**All patients** **(n = 860)**	**Good outcome** **(n = 327)**	**Poor outcome** **(n = 533)**	*P value
pH	7.4 (6.8-7.7) [63]	7.4 (7.1-7.6) [25]	7.4 (6.8-7.7) [38]	0.258^m^
PCO_2_ (mmHg)	36 (16.5-70.7) [63]	36.6 (16.5-55.2) [25]	35.7 (19-70.7) [38]	0.026*^mꝭ^
PO_2_ (mmHg)	102.4 (30.8-545.9) [80]	101.9 (42.2-450.4) [27]	102.7 (30.8-545.9) [53]	0.918^m^
SO_2_ (%)	96.9 (47-99.9) [73]	96.9 (76.6-99.9) [25]	96.9 (47-99.9) [48]	0.569^m^
Lactate (mmol L^-1^)	1.6 (0.5-20.8) [70]	1.7 (0.7-9.3) [26]	1.6 (0.5-20.8) [44]	0.401^m^
HCO_3_^-^ (mmol L^-1^)	20.8 (6.5-32.4) [63]	20.7 (9.3-29.7) [25]	20.8 (6.5-32.4) [38]	0.849^m^
Base excess (mmol L^-1^)	-4 (-26.1-8.1) [63]	-4.3 (-19.7-5.9) [25]	-3.8 (-26.1-8.1) [38]	0.060^ mꝭ^
White blood cell count (×10^3^/µL)	10.7 (0.5-33.6)	10.7 (0.5-33.5)	10.7 (1.2-33.6)	0.419^m^
Haemoglobin (g dL^-1^)	11.3 (4.7-17.7)	11.5 (7.2-16.6)	11.3 (4.7-17.7)	0.035*^m^
Haematocrit (%)	35±5.5	35±5.3	34.7±5.5	0.029*^t^
Mean corpuscular volume (fL)	88.6 (59.4-106.9)	88.7 (61.2-105.1)	88.3 (59.4-106.9)	0.319^m^
Mean corpuscular haemoglobin (pg)	29 (0-38.1)	28.8 (18.9-35.6)	29.1 (0-38.1)	0.496^m^
Mean corpuscular haemoglobin concentration (g dL^-1^)	32.5 (27.6-36.2)	32.6±1.3	32.5±1.3	0.178^t^
Platelet count (×10^3^/µL)	230 (13-977)	231 (39-977)	229 (13-565)	0.417^m^
Mean platelet volume (fL)	10.3 (8.3-111) [14]	10.2 (8.3-81) [5]	10.3 (8.3-111) [9]	0.474^m^
Plateletcrit (%)	0.2 (0-1.2) [14]	0.2 (0.1-1.2) [5]	0.2 (0-1.2) [9]	0.666^m^
Neutrophil count (×10^3^/µL)	8.3 (0.2-30)	8.5 (0.2-26.6)	8.2 (0.7-30)	0.429^m^
Lymphocyte count (×10^3^/µL)	1.4 (0.2-18.1)	1.5 (0.2-5.1)	1.4 (0.2-18.1)	0.009*^t^
Monocyte count (×10^3^/µL)	0.6 (0-3.7)	0.6 (0-2.4)	0.6 (0-3.7)	0.642^m^
Eosinophil count (×10^3^/µL)	0 (0-6.5)	0.1 (0-1)	0 (0-6.5)	0.049*^m^
Basophil count (×10^3^/µL)	0 (0-1.1)	0 (0-1.1)	0 (0-0.3)	0.207^m^
Platelet distribution width (%)	11.4 (0.1-98) [14]	11.4 (0.1-98) [5]	11.4 (7.5-22.1) [9]	0.970^m^
Glucose (mg dL^-1^)	144 (66-568)	145 (68-568)	144 (66-519)	0.691^m^
Creatinine (mg dL^-1^)	0.8 (0.1-7.7)	0.7 (0.3-7.7)	0.8 (0.1-6.8)	0.004*^m^
Estimated glomerular filtration rate (mL min^-1^ 1.73 m^2^)	90 (7-201)	92 (8-151)	87 (7-201)	0.004*^m^
Blood urea nitrogen (mg dL^-1^)	15 (1.1-137)	14 (4-64)	16 (1.1-137)	<0.001*^m^
C-reactive protein (mg L^-1^)	8 (0-510)	7.8 (0.2-341)	8.4 (0-510)	0.044*^m^
Aspartate aminotransferase (U L^-1^)	23 (7.6-2360) [14]	22.8 (8-974) [6]	23 (7.6-2360) [8]	0.671^m^
Alanine aminotransferase (U L^-1^)	16 (5-1164) [22]	16 (5-329) [6]	15 (5-1164) [16]	0.692^m^
Lactate dehydrogenase (U L^-1^)	220.5 (98-3237) [8]	215 (98-3237) [5]	222 (102-2949) [3]	0.119^m^
Albumin (g dL^-1^)	3.4 (1.2-5)	3.5 (1.6-4.7)	3.4 (1.2-5)	0.031*^m^
Total protein (g dL^-1^)	5.7 (2.3-8.8)	5.8 (2.6-8.8)	5.7 (2.3-8.8)	0.116^m^
Total bilirubin (mg dL^-1^)	0.5 (0.1-8.7)	0.6 (0.1-2.9)	0.5 (0.1-8.7)	0.259^m^
Direct bilirubin (mg dL^-1^)	0.2 (0-4.4) [6]	0.2 (0-1.6) [2]	0.2 (0-4.4) [4]	0.829^m^
Calcium (mg dL^-1^)	8.3 (5.7-10.9)	8.4 (6.4-10.9)	8.3 (5.7-10.7)	0.097^m^
Magnesium (mg dL^-1^)	1.9 (0.1-3.3)	1.9 (1-3.3)	1.9 (0.1-3.2)	0.553^m^
Sodium (mmol L^-1^)	139 (123-167)	139 (124-155)	139 (123-167)	0.612^m^
Potassium (mmol L^-1^)	4.2 (2.5-6.8) [5]	4.2 (2.5-5.9) [2]	4.2 (2.7-6.8) [3]	0.512^m^
Amylase (U L^-1^)	55 (3.7-960)	55.5 (13-417)	54.6 (3.7-960)	0.999^m^
Prothrombin time (s)	9.8 (7.6-78.3)	9.7 (7.6-18.2)	9.8 (8.3-78.3)	0.016*^m^
Activated partial thromboplastin time (s)	26.4 (16.4-117) [38]	25.9 (16.4-81.2) [9]	26.6 (17.1-117) [29]	0.070^m^
International normalised ratio (unitless)	1.1 (0.8-8.2)	1.1 (0.8-5.1)	1.1 (0.9-8.2)	<0.001*^m^
**Systemic inflammatory and metabolic indices**				
Neutrophil-to-lymphocyte ratio	5.9 (0.4-69.3)	5.5 (0.9-39.8)	6.2 (0.4-69.3)	0.155^m^
Platelet-to-lymphocyte ratio	161.5 (9.3-1844.4)	148.4 (33.9-1644)	174.1 (9.3-1844.4)	0.033*^m^
Monocyte-to-lymphocyte ratio	0.4 (0-4)	0.4 (0-2)	0.4 (0-4)	0.028*^m^
Systemic immune-inflammation index	1348 (52-30500)	1228 (235-13270)	1384 (52-30500)	0.364^m^
Pan-immune-inflammation value	736.2 (0-22569.6)	703.6 (0-17196.6)	749.1 (0-22569.6)	0.351^m^
C-reactive protein-to-albumin ratio	2.4 (0-188.9)	2.4 (0.1-126.3)	2.5 (0-188.9)	0.033*^m^
C-reactive protein-to-lymphocyte ratio	5.8 (0-1642.3)	4.8 (0.1-1075.3)	6.4 (0-1642.3)	0.022*^m^
Mean platelet volume-to-platelet ratio	0 (0-0.8)	0 (0-0.2)	0.1 (0-0.8)	0.232^m^
Neutrophil-lymphocyte-platelet ratio	0 (0-1.2)	0 (0-0.3)	0 (0-1.2)	0.147^m^
Lymphocyte-to-monocyte ratio	2.6 (0-46.8)	2.8 (0-22)	2.5 (0-46.8)	0.033*^m^
Blood urea nitrogen-to-albumin ratio	4.7 (0.3-76.1)	4.3 (1.1-21.2)	5 (0.3-76.1)	<0.001*^m^
Neutrophil-to-albumin ratio	2.5 (0.1-14.9)	2.6 (0.1-9.2)	2.5 (0.3- 14.9)	0.928^m^
Albumin-to-creatinine ratio	4.2 (0.4-23.6)	4.5 (0.6-10.3)	4.1 (0.4-23.6)	0.001*^m^

Data are presented as mean ± standard deviation or median (minimum-maximum). Missing data are indicated as [n]. ^t^: Student’s t test, ^m^: Mann-Whitney U test. *: *P* value <0.05 was considered statistically significant, ꝭ: Variable sensitive to missing data

**Table 4. Univariable and Multiple Analyses of Factors Associated with Short-term Poor Outcomes in PACU Patients:** 

**-**	**Groups**		**Total** **(n = 860)**	**Univariable**		**Multiple**	
	**Good outcome (n = 327)**	**Poor outcome (n = 533)**		**OR (95% CI)**	*P value	**OR (95% CI)**	*P value
Age (years)	67 (21-92)	69 (18-98)	68 (18-98)	1.006 (0.997-1.016)	0.186	-	-
**ASA scores**							
ASA I	5 (1.5)	5 (0.9)	10 (1.2)	Reference		-	-
ASA II	90 (27.5)	114 (21.4)	204 (23.7)	1.267 (0.356-4.511)	0.715	-	-
ASA III	213 (65.1)	331 (62.1)	544 (63.3)	1.554 (0.445-5.432)	0.490	-	-
ASA IV	18 (5.5)	74 (13.9)	92 (10.7)	4.111 (1.074-15.737)	0.039*	-	-
ASA V	1 (0.3)	9 (1.7)	10 (1.2)	9.000 (0.809-100.139)	0.074	-	-
Inotrope requirement at PACU admission	9 (2.8)	59 (11.1)	68 (7.9)	4.398 (2.15-8.996)	<0.001*	2.785 (1.320- 5.875)	0.007*
Erythrocyte suspension transfusion	37 (11.5) [4]	91 (17.2) [4]	128 (15) [8]	1.606 (1.066-2.42)	0.024*	-	-
Fresh frozen plasma transfusion	16 (5) [4]	51 (9.6) [4]	67 (7.9) [8]	2.047 (1.147-3.655)	0.015*	-	-
**Airway status on PACU admission**					**-**	-	-
Extubated	281 (85.9)	409 (76.7)	690 (80.2)	Reference		-	
Intubated	37 (11.3)	111 (20.8)	148 (17.2)	2.061 (1.379-3.081)	<0.001*	-	
Tracheostomized	9 (2.8)	13 (2.4)	22 (2.6)	0.992 (0.419-2.353)	0.986	-	
**Laboratory parameters and systemic inflammatory-metabolic indices**							
Haemoglobin (g dL^-1^)	11.6±1.8	11.3±1.9	11.4±1.9	0.913 (0.847-0.984)	0.017*	-	
Haematocrit (%)	35.5±5.3	34.7±5.5	35±5.5	0.972 (0.948-0.997)	0.029*	-	-
Mean corpuscular volume (fL)	87.7±6.3	88.3±6.4	88.1±6.3	1.014 (0.993-1.037)	0.199	-	
Mean corpuscular haemoglobin concentration (g dL^-1^)	32.6±1.3	32.5±1.3	32.5±1.3	0.931 (0.838-1.033)	0.178	-	-
Creatinine (mg dL^-1^)	0.8±0.5	1±0.7	0.9±0.7	1.665 (1.237-2.242)	0.001*	-	-
Estimated glomerular filtration rate (mL min^-1^ 1.73 m^2^)	88.1±24.5	81.7±29.6	84.1±27.9	0.992 (0.987-0.997)	0.001*	-	
Blood urea nitrogen (mg dL^-1^)	16.1±8	20.4±14.3	18.8±12.5	1.036 (1.021-1.052)	<0.001*	-	
C-reactive protein (mg L^-1^)	26.9±46.1	42.5±69.4	36.6±62	1.005 (1.002-1.008)	0.001*	-	
Albumin (g dL^-1^)	3.4±0.6	3.2±0.7	3.3±0.6	0.719 (0.576-0.899)	0.004*	-	-
Total protein (g dL^-1^)	5.6±0.9	5.5±1	5.6±1	0.861 (0.748-0.991)	0.037*	-	
Total bilirubin (mg dL^-1^)	0.7±0.4	0.8±0.8	0.7±0.7	1.212 (0.964-1.523)	0.100	-	
Direct bilirubin (mg dL^-1^)	0.3±0.2 [2]	0.3±0.4 [4]	0.3±0.3 [6]	1.615 (0.969-2.692)	0.066^¥^	-	-
Calcium (mg dL^-1^)	8.3±0.6	8.2±0.7	8.2±0.7	0.808 (0.659-0.99)	0.040*	-	-
Amylase (U L^-1^)	67±46.7	72.9±71.4	70.7±63.2	1.002 (0.999-1.004)	0.184	-	
PCO_2_ (mmHg)	36.7±5.7 [54]	36.1±6.6 [13]	36.3±6.2 [67]	0.985 (0.962-1.007)	0.183^¥^	-	-
SO_2_ (%)	96.1±2.8 [54]	95.7±4.6 [14]	95.8±4 [68]	0.971 (0.933-1.011)	0.154^¥^	-	-
Base excess (mmol L^-1^)	-4.4±3.5 [67]	-3.9±4.9 [23]	-4.1±4.5 [90]	1.026 (0.993-1.059)	0.125^¥^	-	-
Activated partial thromboplastin time (s)	27±5.6 [9]	28±8.1 [29]	27.6±7.2 [38]	1.024 (1.000-1.049)	0.046* **^ꝭ^**	-	-
Prothrombin time (s)	9.9±1.1	10.5±3.6	10.2±2.9	1.198 (1.076-1.334)	0.001*	-	-
International normalised ratio (unitless)	1.1±0.3	1.2±0.4	1.2±0.4	8.938 (3.873-20.631)	<0.001*	-	-
Neutrophil-to-lymphocyte ratio	7.4±6.1	8.5±8.1	8.1±7.4	1.021 (1.000-1.042)	0.045*	-	-
Platelet-to-lymphocyte ratio	197.2±158.1	225.7±194.8	214.9±182.2	1.001 (1.000-1.002)	0.028*	-	-
Monocyte-to-lymphocyte ratio	0.5±0.5	0.5±0.5	0.5±0.5	1.836 (1.263-2.671)	0.001*	1.668(1.118-2.488)	0.002*
C-reactive protein-to-albumin ratio	9±16.2	16.8±30.6	13.8±26.4	1.015 (1.007-1.022)	<0.001*	-	-
C-reactive protein-to-lymphocyte ratio	29.9±79.8	61±140.8	49.1±122.2	1.003 (1.001-1.005)	<0.001*	-	-
Blood urea nitrogen-to-albumin ratio	4.9±2.6	7±6.5	6.2±5.5	1.121 (1.075-1.169)	<0.001*	1.100(1.052-1.150)	<0.001*
Albumin-to-creatinine ratio	4.7±1.7	4.2±2.1	4.4±2	0.896 (0.833-0.963)	0.003*	-	-
ln (mean platelet volume/platelet)	-3.1±0.4	-3.1±0.5	-3.1±0.5	1.305 (0.958-1.777)	0.092	-	-
ln (neutrophil to lymphocyte, platelet ratio)	-3.7±0.8	-3.5±0.9	-3.6±0.8	1.199 (1.009-1.425)	0.039*	-	-
Constant	-	-	-	-	-	1.164 (0-0)	0.544

Cox & Snell R square=0.063; Nagelkerke R square=0.085; Accuracy=0.622. Data are presented as number (%) and mean ± standard deviation. Missing data are indicated as [n],  *: *P* value <0.05 was considered statistically significant, ^ꝭ^: Variable sensitive to missing data, ^¥^: Not sensitive to missing data, PACU, post-anaesthesia care unit; ASA, American Society of Anesthesiologists; OR, odds ratio; CI, confidence intervals

**Table 5. Receiver Operating Characteristic Analysis of MLR and BAR for Predicting Poor Short-term Clinical Outcomes:** 

**-**	**MLR**	**BAR**
**Cut-off value**	≥0.375	≥4.605
**AUC (95% CI)**	0.545 (0.506-0.584)*	0.582 (0.544-0.619)^¥^
**Sensitivity (95% CI)**	53.3 (49-57.6)	54.4 (50.1-58.7)
**Specificity (95% CI)**	53.5 (48-59)	54.1 (48.6-59.6)
**Positive predictive value (95% CI)**	65.1 (61.9-68.3)	65.9 (62.7-69)
**Negative predictive value (95% CI)**	41.3 (38-44.6)	42.1 (38.9-45.5)
**Accuracy (95% CI)**	53.4 (50-56.8)	54.3 (50.9-57.7)

Data are presented as estimates with 95% confidence intervals. The optimal cut-off value was determined based on the threshold that minimised the absolute difference between sensitivity and specificity

*: *P* < 0.001. ¥: *P* < 0.001, AUC, area under the curve; CI, confidence interval; MLR, monocyte-to-lymphocyte ratio; BAR, blood urea nitrogen-to-albumin ratio
